# Feeble Females

**Published:** 1875-02

**Authors:** 


### FEEBLE FEMALES.

Feebleness appears to be the natural inheritance of American women. The females of the human race on this continent are fragile flowers as compared with all classes of European women. They fade earlier, and die out young after some years of general or local misery. This should not be so, and need not be, if our people were even moderately intelligent in matters pertaining to physical well-being. The feebleness of our women cannot be attributed to climatic influences, for the continent affords every variety of climate, and in no portion of it can woman compare in health and strength with the rosy English, the elastic French, or the substantial German females. This fact is universally admitted, and it is the part of wisdom for us to endeavor to learn the cause of this physical degeneracy on the part of the mothers and daughters of our race.

There are many reasons for this feebleness on the part of our women, but it is safe to say that there is not one which cannot be obviated to a great extend by themselves, if not *for* themselves at least for their daughters. The great evils from which the sex suffers, with us, is in overwork and underwork.

Thousands and hundreds of thousands of women in America are overworked. This is true almost universally of farmers' wives. Their work is absolutely never done till the grave covers them. Up early and late, they drag around weary limbs all day to find restless couches when the evening is well advanced. Almost their only real times of rest, complete and absolute, are during the brief and oft-repeated periods of maternity, during which the strain upon the system is even greater than during their daily routine of slavery. Upon rising from the couch after the far too brief period of confinement, the daily drudgery must instantly recommence. Heavy burdens must be carried, exhausting labor must be undertaken, not an hour of the day must pass unemployed. "Mother" must furnish the willing hands and the sympathetic heart for the whole family. "Mother" must supply the brains, without which the household would stand still. "Mother" must work hard all day, and perhaps sit up all night, or suffer broken rest, because of the illness of the husband or child. So much for the overwork, which wears out the mothers, in body and in mind, and compels them to give to the world feeble offspring, who, if girls, are destined, it may be, to a like fate, and who will thus do their part in perpetuating the evils of which we speak.

And what of the underwork? That is the sin of the fortunate, just as overwork is the sin of those who deem themselves unfortunate in not being abundantly endowed with worldly goods. The crime of overwork is the crime of such as are earnest to save; the crime of underwork is the crime of the indolent, who are eager to spend, and who look upon nothing with the abhorrence which they do upon exertion. There are multitudes of women among the well-to-do classes who are enfeebled by sheer laziness. These are the delicate creatures who never walk, who sit in doors all winter, because it is too cold, and who lounge about all summer because it is too warm. They sometimes go out, but it must be in a carriage, or any way save on foot. They are never so happy as when eating, or drinking, or dancing, or visiting (in a carriage), or at some crowded and unwholesome place of amusement. These lovely creatures are great patrons of the doctors; they are, of course, always complaining.

They take naturally to wine and all stimulants, because they are heavy and inert, and all their functions are torpid, and need "toning up."

These Feeble Females die young, but not as young as they should. It is sad to know that before they lay down their altogether useless lives they not unfrequently give to the world one or more specimens of the same kind. The useless type of the Feeble Female is thus kept up, to the demoralization of society and the gradual but sure deterioration of the race.

How shall these two classes of errors be righted? The first must be rendered impossible by a tidal wave, like that which made black slavery intolerable, and the last must be annihilated by the spread of knowledge and intelligence. It must be made an odious offence to overwork the wife and mother at the very period when she should have care and affection and relief from onerous duty of body and brain; and it must be recognised as the supremest of follies to live for fashion and frivolity. Society in the country must be brought up to woman's standard, and society in the city must be placed upon the solid basis which prevails among the most intelligent classes of Europe, a foundation of purity and integrity such as will make women fonder of their homes and home duties than the endless whirl of dissipation and excitement which the women of the day indulge in to so great an extent. If our city women were to think less of dress, of tight lacing, of late suppers and balls, and attend more closely to home duties and home pleasures, blended with life in the open air, we should have renewed hopes for the coming generations.

